# Dosimetric impact of hollow intraoral stents for head and neck cancer radiotherapy: A phantom study

**DOI:** 10.1002/acm2.14101

**Published:** 2023-07-21

**Authors:** Hongbing Song, Jian Hu, Junxiang He, Guangdong Ma, Lan Cheng, Xiangpan Li

**Affiliations:** ^1^ Department of Radiotherapy Renmin Hospital of Wuhan University Wuhan Hubei China; ^2^ Department of Radiology Union Hospital TongJi Medical College Huazhong University of Science and Technology Wuhan Hubei China

**Keywords:** head and neck cancer, intraoral stent, OSLD, TPS

## Abstract

**Purpose:**

To investigate the dosimetric impact of the calculation boundaries and dose calculation algorithms of radiotherapy in head and neck cancer patients with an opened oral cavity connected to the exterior by a hollow intraoral positioning stent.

**Methods and Materials:**

A homemade silicone phantom with an opened oral cavity was placed in a CIRS head phantom to model head and neck cancer patients with a hollow intraoral positioning stent. 3D‐CRT plans were designed on CT images of the phantom in Monaco and Pinnacle^3^ treatment planning systems (TPSs) with the same beam parameters. The default boundary and manually extrapolated boundary were both adopted in these two TPSs to explore the dosimetric impact on treatment plans. The nanoDot™ optically stimulated luminescence dosimeters (OSLDs) were chosen to measure the planned dose surrounding the oral cavity of the head phantom after calibration.

**Result:**

The doses in the air cavity and two measuring points at the joint area were dramatically changed from 0.0, 92.4 and 148.8 cGy to 177.8, 244.2 and 244.1 cGy in Monaco after adopting the extrapolated boundary. While the calculated doses at the same place were changed from 61.2, 143.7 and 198.3 cGy to 175.4, 234.7 and 233.2 cGy in Pinnacle^3^ with a similar calculation boundary. For the Monaco TPS, the relative errors compared to the OSLD measured doses were 2.94 ± 1.93%, 0.53 ± 8.64%, 2.65 ± 1.87% and 3.93 ± 1.69% at 4 measuring positions. In contrast, the relative errors 4.03 ± 1.93%, 4.85 ± 8.64%, 7.61 ± 1.87% and 5.61 ± 1.69% were observed in Pinnacle^3^.

**Conclusion:**

The boundary setting of an opened oral cavity in TPSs has a significant dosimetric impact on head and neck cancer radiotherapy. An extrapolated boundary should be manually set up to include the whole oral cavity in the dose calculation domain to avoid major dose deviations.

## 1 INTRODUCTION

Intraoral positioning stents are commonly applied in head and neck cancer radiotherapy to protect the healthy tissues surrounding tumors from unnecessary radiation and to minimize radiation‐induced oral mucositis.^1^ **^,^** ^2^ These stents are usually hollow to maintain the airway unobstructed and keep the mouth open during radiotherapy treatment.^3^, ^4^, ^5^, ^6^, ^7^, ^8^ Due to their special structures and component materials, though potentially uncomfortable for patients, intraoral stents provide significant dose sparing to the oral cavity.

In most commercial radiotherapy TPSs, a special contour of the human body, like ‘BODY’ in Eclipse (Varian Medical Systems, Palo Alto, CA, USA) and ‘patient’ in Monaco (Elekta CMS, Maryland Heights, MO, USA), must be defined to set the boundary of the computational domain before dose calculation. It can be generated easily by TPSs according to the CT value differences between the human body and air. However, the automatically‐generated boundary usually indents the inner wall of the oral cavity in cancer patients with an intraoral stent due to the hollow oral stent. Therefore, if the automatically generated boundary is adopted during dose calculation, everything outside the boundary is treated as nothing, even though the oral cavity is filled with air. Medical physicists debate whether or not to manually extrapolate the boundary to the body outline during treatment planning, which affects the actual radiotherapy dose delivered to the tumor and adjacent tissues of the oral cavity. Directly simulating the photon beam in a TPS is the simplest way to find the answer. However, different dose algorithms may produce varying results near an air cavity.^9^, ^10^ The Monte Carlo method is the most trusted algorithm owing to its high fidelity in presenting the dosage distribution in an inhomogeneous medium, which has charged particle disequilibrium.^11^, ^12^ However, the fast Monte Carlo method commonly adopted by commercial TPSs may also have lower precision in low‐density materials like air.^13^ Therefore, combining TPS simulation results with precise measurements provides a more reliable method in practical cases.

Many researchers have explored the radiation dose near or in an enclosed air cavity by using different dosimeters, including radiochromic film, alanine dosimeter probes, thermoluminescent dosimeter (TLD), and the newly‐developed OSLD, which is very convenient and has high efficiency.^9^, ^10^, ^14^, ^15^, ^16^ Unfortunately, few studies paid attention to the dosage around an opened air cavity, like the oral cavity or the nasal cavity, which is directly connected to the outside. This study investigated the dosimetric impact of the indented and extrapolated boundaries for a 3D‐CRT treatment plan and the dosage differences in various commercial TPSs. In order to verify these calculated results, OSLDs were utilized to measure the radiation dose during the treatment in a homemade silicone phantom. The findings of this study will help medical physicists determine a more appropriate calculation boundary in daily treatment planning involving hollow intraoral positioning stents. Furthermore, a more precise treatment plan will improve the protection of the surrounding healthy tissues.

## 2 MATERIAL AND METHODS

In this study, 32 nanoDot™ OSLDs (Landauer Inc., Glenwood, IL, USA) were incorporated to measure the irradiation dose in all phantoms. The OSLDs were calibrated and verified before the final measurement. Previous studies have reported that the angular dependence response of the dosimeter can be ignored when the plane of the OSLD is parallel to the beam direction.^17^, ^18^ Therefore, the OSLDs were all kept with their disc parallel to the beam during the calibration and measurement.

### 2.1 Determination of individual sensitivity factor (ISF)

**FIGURE 1:**
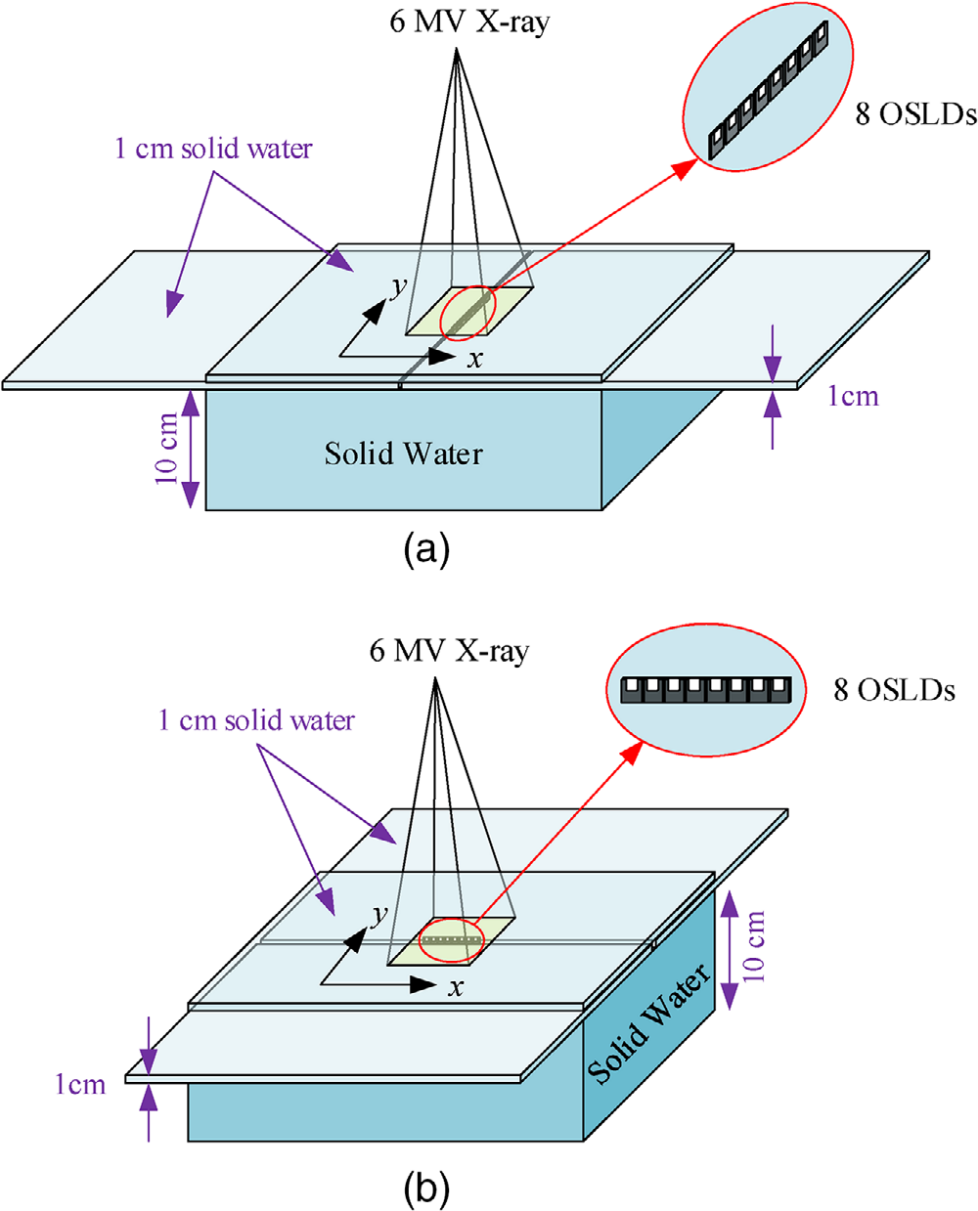
Calibration of all the 32 OSLDs with 6 MV photon beam in four separate irradiations/measurements. (a) 8 OSLDs were placed along the direction of the beam between two 1 cm thick solid water blocks along the *y* direction of the radiation field, (b) 8 OSLDs were placed along the direction of the beam between two 1 cm thick solid water blocks along the *x* direction of the radiation field.

Firstly, all 32 screened and annealed nanoDot™ OSLDs were irradiated by a 6 MV photon beam in a 10 × 10 cm^2^ field size using the Varian Clinac iX linear accelerator system (Varian Medical Systems, Palo Alto, CA, USA) with a dose rate of 600 MU/min in the EASY SLAB solid water phantom (LAP GmbH Laser Applikationen, Lueneburg, Germany). The source‐skin distance (SSD) was set to 100 cm. The sensitive area center of all the OSLDs was placed at a depth of 1.5 cm (e.g., the *d*_max_ point for 6 MV photon beam in water). A 10 cm thick solid water block was laid beneath the OSLDs to provide sufficient backscatter. For the irradiation, eight OSLDs were placed between two 1 cm thick solid water blocks along the *y* direction of the radiation field and were covered by a 1 cm thick solid water block. Subsequently, 8 OSLDs were irradiated along the *x* direction with the same method, as illustrated in Figure 1a and b. For the size of nanoDot™ OSLD is 10 mm × 10 mm × 2 mm, when it was set parallel to the beam, the center of the sensitive area in OSLD was about 1.5 cm deep from the solid water surface. In each irradiation, the linear accelerator beamed 100 cGy towards the solid water block. The OSLD measurements were captured by a microStar II OSLD reader (Landauer, Glenwood, IL, USA) after 15 min to allow for the significant signal fade of OSLDs in the first few minutes.^19^ All OSLDs were irradiated only once and then annealed again before their reuse.

Secondly, the ISF of every OSLD was calculated based on the measured count of each OSLD $M_{c}$ in the previous step and their average count $M_{a}$ using the equation $\mathrm{ISF}\;=M_{c}/M_{a}\;$.^20^, ^21^ Each OSLD may have a varying response under the same radiation dose depending on the manufacturing process. Applying ISF to every OSLD can improve the uniformity and predictability of the measurement results.

### 2.2 Absolute dose calibration

The nanoDot™ OSLD showed a linear response between counts and irradiation dose below 200 cGy while exhibiting a supra‐linear relationship above 200cGy, which was attributed to the extra luminescence from deeper electron traps in the detector during irradiation.^19^, ^22^ In this study, 4 randomly selected OSLDs were used to calibrate the response relationship between OSLD counts and irradiated dose. These 4 OSLDs were also placed at a depth of 1.5 cm in a 10 × 10 cm^2^ field. Two of them were placed along the *y* direction of the radiation field, and the other two were arranged along the *x* direction. The linac beamed 50 cGy to these four OSLDs and was increased step by step from 50 to 300 cGy. The net counts of the four OSLDs were calculated for each irradiation by subtracting the readings prior to irradiation from the post‐irradiation measurements. The average responses and standard deviations were calculated from the OSLD measurements, and linear and quadratic polynomial fitting of these dose points were performed. Following the above process, the precise relationship between irradiated doses and OSLD measurements was successfully determined.

During our quality control test, we found that the deviation of the angular response of nanoDot™ OSLD after calibration only ranged between −1.79%−1.65%. Therefore, we did not consider the angular dependence of OSLDs in calibration in this study due to its negligible effect when the OSLD disc is parallel to the irradiation beam.^17^, ^18^

### 2.3 Verification in the slab phantom

In order to verify the measurement accuracy of the calibrated nanoDot™ OSLDs, a 30 cm thick pile of EASY SLAB solid water blocks was utilized as the phantom. Four OSLDs were adopted to measure the dose at depths of 1.0 cm, 1.5 cm, 2.0 cm, 3.0 cm, 4.0 cm, 5.0 cm and 6.0 cm. Two OSLDs were arranged along the *y* direction and the other two OSLDs along the *x* direction of the irradiation field. The gantry angle of linac was maintained at 0°. OSLDs were centered in a 10 × 10 cm^2^ irradiation field with SSD set to 100 cm, and 100 cGy was beamed from the linac at a dose rate of 600 MU/min. A TW30013 Farmer‐type ion chamber (PTW, Freiburg, Germany) was placed at the same depth to measure the beam dose for comparison. The ion chamber was connected to a UNIDOS E dosimeter (PTW, Freiburg, Germany) by an electric cable. The irradiations and measurements were repeated three times at each depth.

The percentage depth dose (PDD) of a 10 × 10 cm^2^ irradiation field size at gantry 0° with the SSD set to 100 cm in a 30 cm × 30 cm × 30 cm virtual water phantom was calculated by using Elekta Monaco V5.40.04 (Elekta CMS, Maryland Heights, MO, USA) with a 1 mm dose grid and 0.5% statistical uncertainty. The density of the water phantom cube was set to 1.045 g/cm^3^ in Monaco TPS to match the real density of the EASY SLAB solid water.

### 2.4 Dose verification in the anthropopathic head phantom

#### 2.4.1 The structure of the home‐made head phantom

**FIGURE 2:**
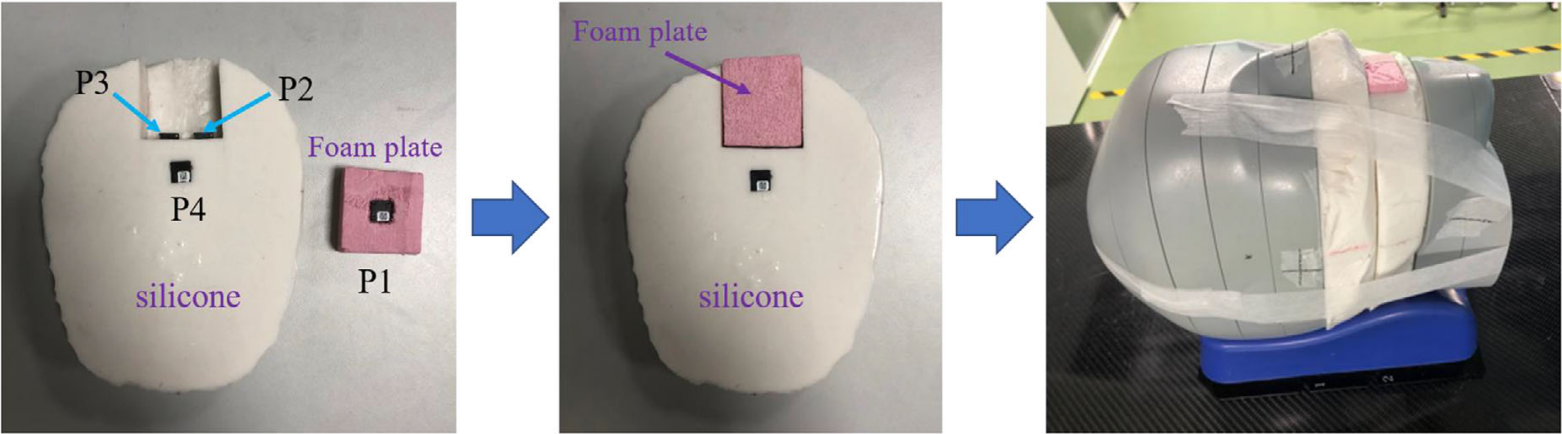
Locations of the 4 nanoDot™ OSLDs in the homemade silicone phantom sandwiched in a CIRS head phantom.

In this study, two slices of the head of an adult female CIRS ATOM^®^ phantom (Computerized Imaging Reference Systems Inc., Norfolk, VA, USA) were replaced by homemade silicone slices (*ρ* = 1.22 g/cm^3^), which had the same outline as the original ones to construct an opened oral cavity simulating a hollow intraoral positioning stent. The assembly process of the new head phantom and the locations of the four OSLDs are illustrated in Figure 2. A piece of cut foam plate with an average density of 0.07 g/cm^3^ was stuffed in the cavity of the silicone phantom to simulate air in the oral cavity connected to the outside and provided a measuring position for OSLD. The foam plate has a density very close to the air (<0.02 g/cm^3^) and it will be easily discarded by TPSs like the hollow intraoral stent when generating the boundary contour of the computational domain. It also can be a typical example of intraoral stent with low density. Two OSLDs were placed at the junction of the cavity and the silicone phantom to reproduce the received dosage of the mucous membrane in the oral cavity during treatment. The last OSLD was located at the center of the phantom, where the planning target volume (PTV) was delineated.

#### 2.4.2 Planning and dosimetric comparison

**FIGURE 3:**
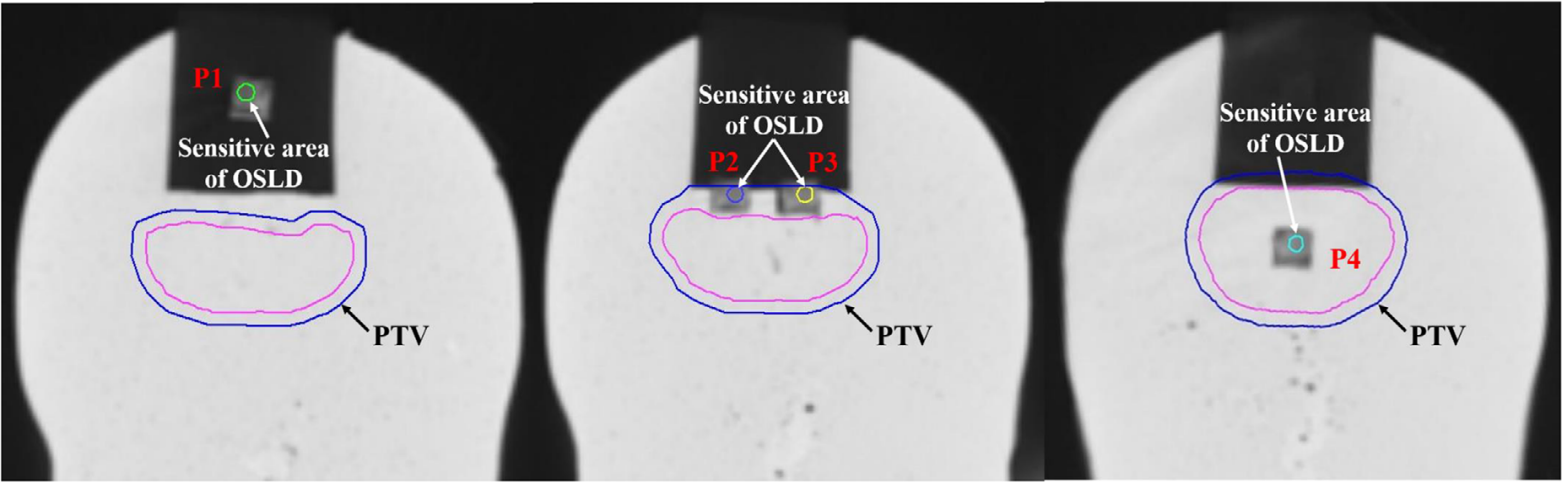
The space locations of four OSLDs and the PTV on CT images.

**FIGURE 4:**
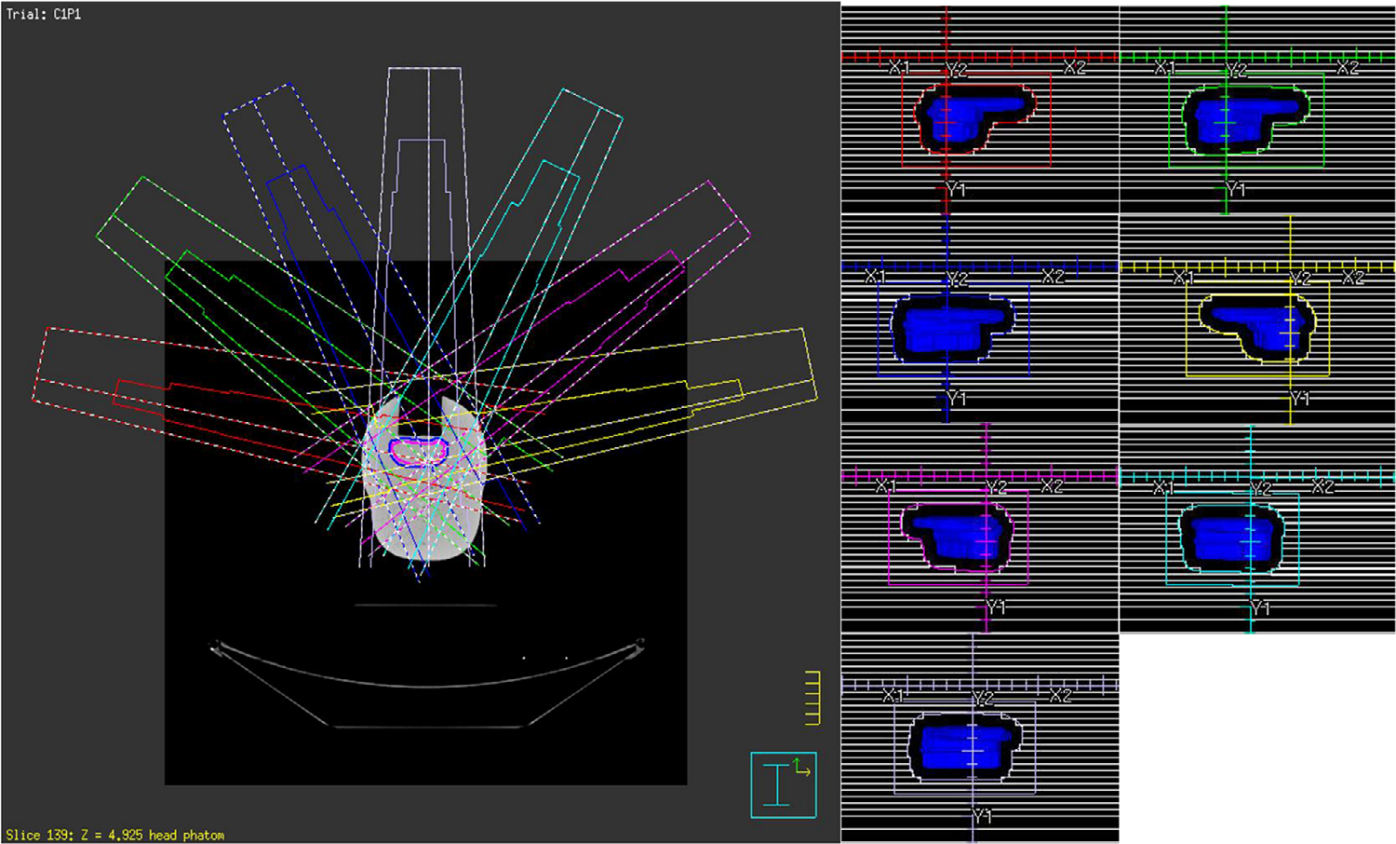
The beam geometry of the seven‐beam 3D‐CRT plan in Pinnacle^3^ TPS.

Four OSLDs were assembled in the homemade silicone phantom, which was sandwiched in the CIRS head phantom during position location and scanned by GE Discovery CT590 RT CT scanner (GE Healthcare, Little Chalfont, UK). The images were acquired at a 1 mm thickness, allowing visual confirmation of the sensitive area of OSLDs. The images were then transferred to Monaco TPS. Subsequently, a PTV was delineated near the air cavity, and the sensitivity area of the four OSLDs was also delineated in the positioning CT images. The space relationship between the four OSLDs and the PTV on CT images was shown in Figure 3. The prescribed dose for a single fraction of the PTV was 220 cGy. In order to avoid small segmented fields, the IMRT and VMAT techniques were not adopted in this study. Instead, two seven‐beam 3D‐CRT plans were designed on Monaco TPS V5.40.04 and Pinnacle^3^ TPS version 9.10 ((Philips Medical Systems, Andover, MA, USA) with the same beam parameters, respectively. The beam angles of the plans in this study were 282°, 308°, 334°, 78°, 52°, 26°and 0° and no angle was below the treatment couch to avoid any attenuation. The beam angles and their field sizes of the plan were illustrated in Figure 4. The Monaco TPS used the Monte Carlo method with a 1 mm dose grid and 0.5% statistical uncertainty to calculate the dose, while the Pinnacle^3^ TPS calculated the dose with the collapsed cone convolution (CCC) algorithm in a 0.1 cm × 0.1 cm × 0.1 cm dose grid. However, both TPSs did not calculate the dose at the air cavity that was directly connected to the exterior. In Monaco TPS, the auto‐generated contour structure named ‘patient’ did not include the air cavity due to its low CT value (<−200 HU). In addition, Pinnacle^3^ also discarded the air cavity as the density was lower than the threshold of 0.6 g/cm^3^. Therefore, an extrapolated ‘patient’ contour, including the edge of the foam plate in the head phantom, was adopted to replace the original ‘patient’ in Monaco TPS to analyze the impact of the different calculation boundaries. In order to minimize the dose deviation resulting from the outside‐patient air threshold density, a very low density of 0.05 g/cm^3^ was set up in Pinnacle^3^ to enlarge the calculation boundary around the oral cavity. The two plans with extrapolated external contours were compared with the previous default ones. The mean doses of the four OSLDs calculated by the two TPSs were considered as the reference value for comparison with the measured dose.

#### 2.4.3 Dose measurement and evaluation

The treatment unit to fulfill the treatment plan was still the Varian Clinac iX linear accelerator system, and the treatment was controlled by the MOSAIQ system (Elekta CMS, Maryland Heights, MO, USA). The head phantom was set up with the aid of the LAP laser system (LAP GmbH Laser Applikationen, Lueneburg, Germany). The irradiations and measurements were repeated three times, and the OSLDs used each time were all screened and annealed. In order to avoid the extra dose from kV cone beam CT (CBCT) on linac, no image‐guided radiotherapy was adopted in this study. The OSLD measurements were still collected 15 min after the irradiation. The measured results were analyzed against the planned dose according to the American Association of Physicists in Medicine Task Group (TG)−119 formalism with $\frac{\mathrm{measured}\;\mathrm{dose}-\mathrm{planned}\;\mathrm{dose}}{\mathrm{prescribed}\;\mathrm{dose}}$ for point doses.^23^

## 3 RESULTS

### 3.1 Calibration results

**FIGURE 5:**
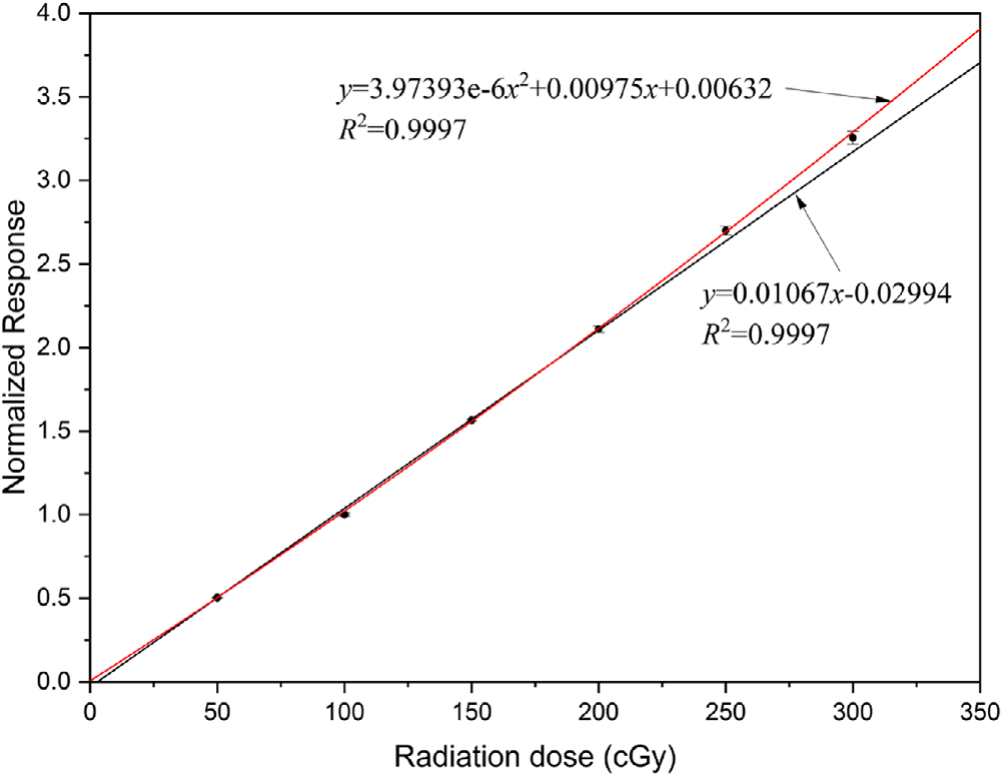
The calibration curve between radiation dose and dose response of nanoDotTM OSLD obtained by using linear fitting (black line) and quadratic polynomial fitting (red line) for 6 MV photon beam.

The calibration curve of nanoDot™ OSLD at range 0∼300 cGy is illustrated in Figure 5. The black line shows a linear fitting between the irradiation dose and counts of OSLDs. An obvious supra‐linear relationship was observed when the irradiation dose exceeded 200 cGy. Therefore, a quadratic polynomial fitting was adopted to correctly reflect the dose response of nanoDot™ OSLDs above 200 cGy, as represented by the red line in Figure 5. The fitting formulas and their R‐squares are also listed in Figure 5.

### 3.2 Verification results in the solid water phantom

**FIGURE 6:**
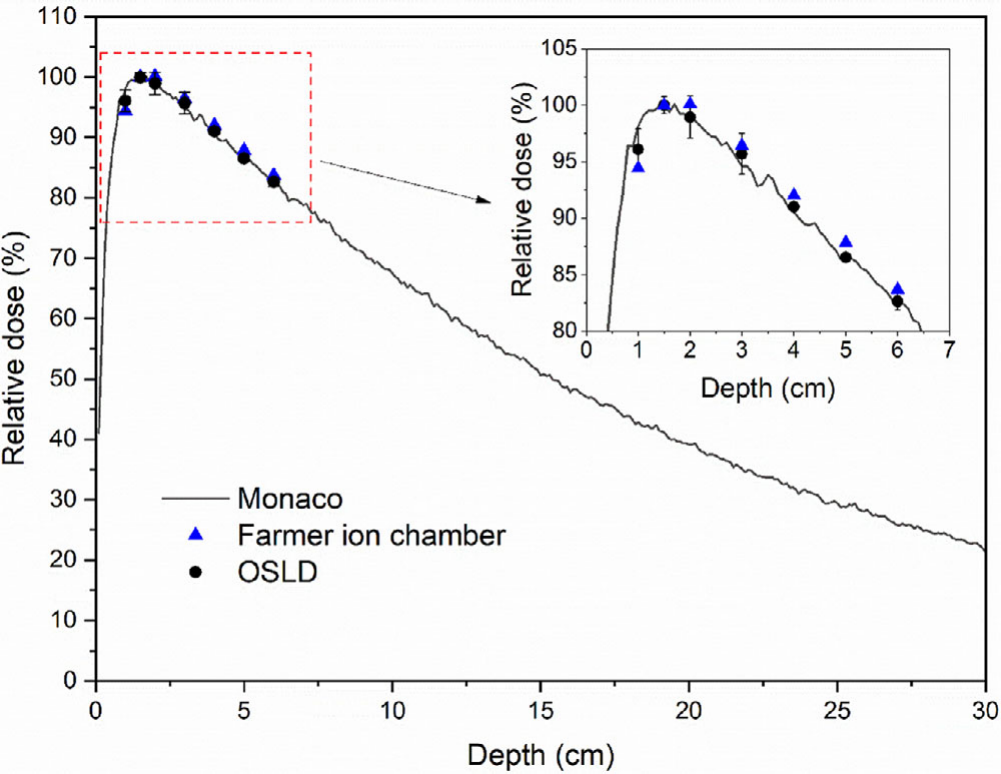
OSLDs and PTW Farmer ionization chamber measurements of PDDs of the 6 MV photon beam irradiated in a 10 × 10 cm^2^ field size in the solid water phantom compared with the Monaco TPS‐simulated results.

The results of OSLDs and ion chamber measurements at a depth of 1.0–6.0 cm were compared to the calculated dose from Monaco TPS, as displayed in Figure 6 and Table 1. As the dosage beamed to the solid water phantom was only 100 cGy per irradiation, the calibration results utilized the linear fitting. Figure 6 shows that the measured results of OSLDs and PTW Farmer ionization chamber closely followed the PDD curve calculated by Monaco TPS. The maximum difference of −2.05% can be observed at a depth of 1.0 cm, whereas the variations were almost below 1.0% at other depths. For the PTW Farmer ion chamber, the maximum deviation of −3.73% was observed at a depth of 1.0 cm, while the differences at other depths were below 2.0%. OSLDs presented more precise measuring results than the Farmer ion chamber when compared with the TPS‐simulated results.

**TABLE 1:** OSLDs and PTW Farmer ionization chamber measurements of PDDs compared with Monaco‐calculated results of 6 MV photon beam irradiated in a 10 × 10 cm^2^ field size.

Depth/cm	Monaco/%	OSLDs/%	diff/%	Ion chamber/%	diff/%
1.0	98.14	96.09	−2.05	94.41	−3.73
1.5	100.00	100.00	–	100.00	–
2.0	98.92	98.94	0.02	100.10	1.18
3.0	94.60	95.69	1.09	96.42	1.82
4.0	90.58	91.00	0.42	92.01	1.43
5.0	86.95	86.53	−0.42	87.85	0.90
6.0	82.14	82.64	0.50	83.69	1.55

### 3.3 Dosage in the head phantom

**FIGURE 7:**
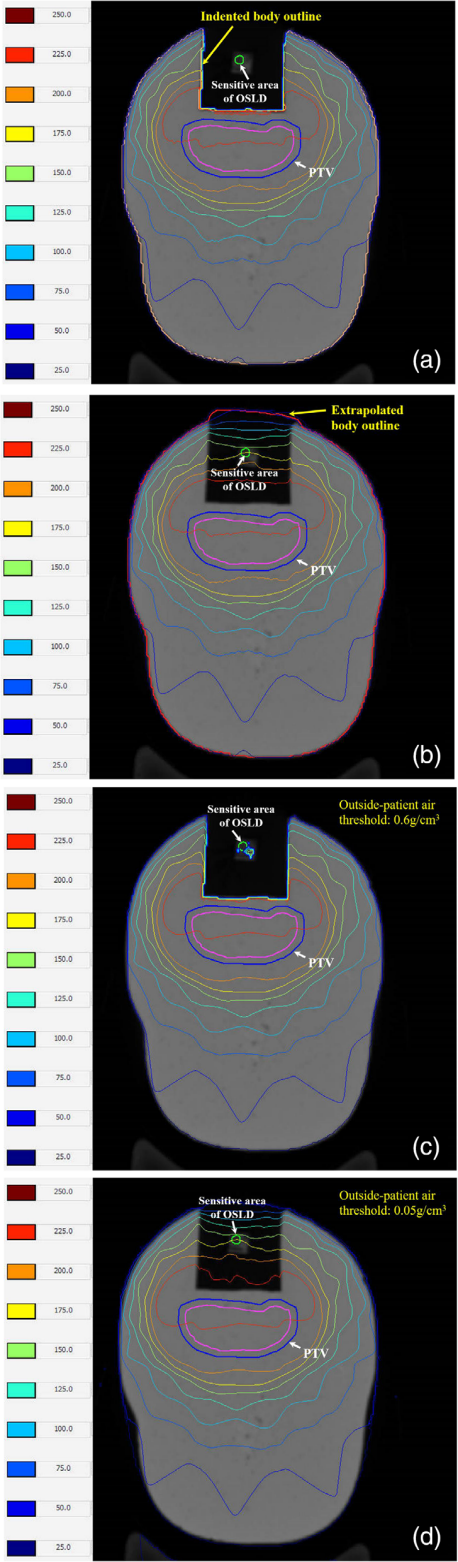
Dosage surrounding the oral cavity in head phantom calculated by Monaco and Pinnacle3. (a) Dose calculated by Monaco with an indented body outline (brown line), (b) Dose calculated by Monaco with an extrapolated body outline (red line), (c) Dose calculated by Pinnacle3 with a 0.6 g/cm^3^ outside‐patient air threshold, (d) Dose calculated by Pinnacle^3^ with a 0.05 g/cm^3^ outside‐patient air threshold.

The dose surrounding the oral cavity in 3D‐CRT plans simulated in Monaco and Pinnacle^3^ with default settings are illustrated in Figure 7a and c, and the plans with extrapolated external contours are presented in Figure 7b and d, respectively. Table 2 presents the calculated doses of OSLDs in the four positions around the oral cavity, and Table 3 compares the deviations of OSLD measured doses to the calculated doses of the treatment plans with extrapolated external contours from Monaco and Pinnacle^3^. In Table 2, $D_{Mo}$ and $D_{Me}$ refer to the calculated doses with an original indent patient boundary and an extrapolated patient boundary in Monaco, respectively. Furthermore, $D_{Po}$ and $D_{Pe}$ refer to the simulated doses with the outside‐patient air threshold set to 0.6 g/cm^3^ and 0.05 g/cm^3^ in Pinnacle^3^, respectively.

**TABLE 2:** Doses in 4 OSLD locations calculated by Monaco and Pinnacle^3^. $D_{Mo}$ and $D_{Me}$ refer to the calculated doses with an original indent patient boundary and an extrapolated patient boundary in Monaco, respectively. $D_{Po}$ and $D_{Pe}$ refer to the simulated doses with the outside‐patient air threshold set to 0.6 g/cm^3^ and 0.05 g/cm^3^ in Pinnacle^3^, respectively.

Dose/cGy	P1	P2	P3	P4
$D_{Mo}$	0.0	92.4	148.8	228.4
$D_{Me}$	177.8	244.2	244.1	226.8
$D_{Po}$	61.2	143.7	198.3	224.1
$D_{Pe}$	175.4	234.7	233.2	223.1

**TABLE 3:** OSLD measured dose differences by locations in head phantom compared to the Monaco and Pinnacle^3^‐calculated results with manually extrapolated boundaries.

	Monaco				Pinnacle^3^			
Location	Mean	SD	Minimum	Maximum	Mean	SD	Minimum	maximum
P1	2.94%	1.93%	0.68%	5.39%	4.03%	1.93%	1.77%	6.48%
P2	0.53%	8.64%	−11.64%	7.45%	4.85%	8.64%	‐7.32%	11.77%
P3	2.65%	1.87%	0.16%	4.68%	7.61%	1.87%	5.11%	9.64%
P4	3.93%	1.69%	2.41%	6.29%	5.61%	1.69%	4.09%	7.97%

As shown in Figure 7, the plans with extrapolated external contours resulted in completely different dose distributions from the original plans. The calculated dose of P1 in Table 2 also numerically indicates this phenomenon. In the original Monaco plan, everything outside the patient boundary was deemed as nothing, so the simulated dose at P1 was zero. In contrast, the original Pinnacle^3^ plan calculated a dose of 61.2 cGy at P1 for the high‐density OSLD components when the outside‐patient air threshold was set to 0.6 g/cm^3^. When an extrapolated patient boundary and a lower air threshold (*ρ* = 0.05 g/cm^3^) were set in Monaco and Pinnacle^3^, respectively, two close doses 177.8 cGy and 175.4 cGy were calculated at P1 with a dose difference of 1.3%. The indented boundary indicated dramatically decreased doses at P2 and P3 in both TPSs, but Pinnacle^3^ was less affected due to its density identification. However, the dose differences of 3.9% and 4.5% were still observed at P2 and P3 between the two plans with extrapolated external contours. As seen in Table 2, the calculation boundary had a lower effect on the dose calculation at P4 in the PTV area in both TPSs. The dose deviations at P4 after modification were only 0.7% and 0.4% in Monaco and Pinnacle^3^, respectively.

As the prescribed dose of the PTV was 220 cGy per fraction, the OSLD measured doses from P1 to P4 were calibrated by using the quadratic polynomial fitting. As seen in Table 3, the differences between OSLD measured doses and Monaco TPS calculated doses at locations P1 to P4 were 2.94 ± 1.93%, 0.53 ± 8.64%, 2.65 ± 1.87% and 3.93 ± 1.69%, respectively. While the relative errors compared to Pinnacle^3^ were 4.03 ± 1.93%, 4.85 ± 8.64%, 7.61 ± 1.87% and 5.61 ± 1.69%, respectively. For the prescribed dose was a constant in this study, the standard deviations (SD) of the dose differences relative to both TPSs calculated by the formula $\frac{\mathrm{measured}\;\mathrm{dose}-\mathrm{planned}\;\mathrm{dose}}{\mathrm{prescribed}\;\mathrm{dose}}$ were the same in Table 3.

## 4 DISCUSSION

In this study, a homemade silicone phantom with an opened oral cavity sandwiched in a CIRS head phantom was utilized to analyze the impact of calculation boundary on the received dosage in head and neck cancer radiotherapy. The nanoDot™ OSLDs were placed around the oral cavity inside the head phantom to measure the actual irradiation dose. The simulation results demonstrated that the boundary setting of the oral cavity in TPSs exerted a great impact on the dosage distribution of the air cavity and air‐tissue joint area. The measured doses in those locations indicated that the hollow oral cavity should be included in the calculation domain, even though it connects to the outside. The air in the oral cavity impacted the dose of head and neck cancer radiotherapy, and changes in the positioning of the intraoral stents and tracheostomy devices have raised concerns.^11^, ^15^, ^24^ Medical physicists also have intuitively pushed out the generated invagination boundary in TPS to obtain a better skin dose distribution when planning for radiotherapy of head and neck cancer patients, although there no measurement results are available to support this behavior.^5^, ^8^, ^25^ The present study filled this gap by demonstrating the validity and necessity of pushing out the cavity boundary in a treatment plan from both planning and practical measurement perspectives.

The Elekta Monaco TPS and Phillip Pinnacle^3^ TPS were adopted in this study to analyze the dosage difference around the opened oral cavity by using the Monte Carlo algorithm and the CCC algorithm, respectively. It is generally assumed that the Monte Carlo algorithm is more precise than the CCC algorithm in an area featuring sharp density changes. In this study, the photon beam backscatter caused by the foam and silicone phantom interface resulted in a steep dose curve near the interface. This phenomenon can be easily predicted by the Monte Carlo algorithm or deterministic grid‐based Boltzmann equation solver (GBBS or the discrete ordinates method), and the CCC algorithm also predicts a precision dose when the field size is large enough.^26^ The simulation results of this study also indicated that the discrepancy between the two TPSs at the joint area was small, namely about 10 cGy or 4.5% of the prescribed dose, when the extrapolated boundary was adopted, as shown in Table 2. However, this discrepancy was significantly larger when the boundary conditions were changed to the default settings. These findings suggest that the calculation boundaries of low‐density areas in TPS exert a significant impact on the final simulation results and should be carefully evaluated. The measured results in Table 3 confirmed that pushing out the calculation boundary of an open oral cavity in TPS before planning provided more accurate irradiation doses. This correction can be easily achieved in Elekta Monaco and Varian Eclipse, but a lower outside‐patient air threshold should be set manually in Pinnacle^3^ to achieve a similar effect for it do not need to define a visible contour of the calculation boundary before dose calculation. In this study, only the 3D‐CRT technique was adopted during planning but the dose impact of hollow intraoral stent for head and neck cancer patients treated with IMRT and VMAT techniques will be investigated in our future work.

The OSLDs utilized play an essential role in this study. The nanoDot™ OSLD is mainly composed of a 5‐mm diameter and 0.2‐mm thickness aluminum oxide doped with carbon (Al_2_O_3_:C) (*ρ* = 1.41 g/cm^3^) and a 10 mm × 10 mm × 2 mm light‐tight plastic holder (*ρ* = 1.03 g/cm^3^).^19^, ^27^ It is the most widely‐available OSLD used in diagnostic radiology and therapeutic radiation oncology.^21^, ^28^, ^29^ The device features a good response and high‐precision measurement of high‐energy photons and electrons. According to Kim et al., the measurement deviation of nanoDot™ OSLD to a known dose can be within ±3.0% when solely using the factory calibration, and ±1.5% standard deviation can be achieved by applying calibration factors if all the OSLDs originate from the same batch.^16^ In general, the nanoDot™ OSLD is accurate within ± 3% for therapeutic doses after calibration.^20^, ^21^, ^24^, ^29^ Our PDD measurement in solid water phantom also was consistent with these studies. The maximum deviation between the simulated dose of Monaco and the measured OSLD dose was −2.05% at a depth of 1.0 cm, and the deviations were almost all below 1.0% in the range of 1.5 cm to 6.0 cm. Due to the supra‐linear characteristic of OSLD response at irradiation doses above 200 cGy, a quadratic polynomial fitting or polynomial fitting of higher order should be adopted to describe such a relationship, resulting in more precise measurements. In the Monaco TPS, the maximum deviation from the measured dose was less than 4.0%, and the average deviation of four locations was 2.51%, which were lower than the maximum deviation of 7.61% and the average deviation of 5.53% in Pinnacle^3^. These results demonstrated that the Monte Carlo algorithm indeed produced a more precise dosage than the CCC algorithm in such conditions. However, we also noticed the deviations at the 4 locations fluctuated and were inconsistent, especially at P2. The SD 8.64% at P2 were even larger than other locations, which presented that the dose repeatability was not good at the foam‐silicone joint area of the head phantom. That may be caused by deviations in the setup of OSLDs in the head phantom and the head phantom itself during irradiation. Although the homemade silicone phantom was obtained by a reverse mold of the original CIRS head phantom, the new phantom cannot be assembled like the original one, and the pits in the homemade phantom may not exactly fit the OSLDs. These gaps may cause minor setup errors during irradiation. In addition, the CBCT image guide was not implemented in this study to avoid additional radiation dose to the OSLDs, and the setup of the head phantom before irradiation was only performed with the help of a cross mark and the laser system in the treatment room, which also introduced setup errors for the final measurement results.

Besides, several limitations that may also give rise to unwanted errors should be mentioned. Firstly, due to the sensitive area of nanoDot™ OSLD not being located at the center of the 1 cm × 1 cm plastic holder plane; it is located at the 0.4 cm × 0.4 cm position. Therefore, the calibration depth was 1.6 cm rather than 1.5 cm in this study, which may introduce some system errors. Secondly, the space between the OSLD sensitive area and the outside plastic holder is full of air, which decreases the attenuation of X‐rays before reaching the sensitive area. Some studies have injected water inside the OSLD to force the air out, but water immersion and subsequent air‐drying result in permanent OSLD degradation and are not recommended.^16^, ^30^ Thirdly, a total of 32 OSLDs were utilized in this study, but they showed divergent measuring results under the same calibration curve. Therefore, OSLDs having a close response curve should be selected to provide more precise measurements. Finally, the angular dependence of OSLD was not considered in this study. That also introduces minor system errors to the final results.

## 5 CONCLUSION

The boundary of the air cavity in an oral positioning stent connected to the outside has a significant dosimetric impact on the oral cavity and the surrounding tissues in head and neck cancer radiotherapy. An extrapolated boundary should be manually set up to include the whole oral cavity in the dose calculation domain to avoid major dose deviations during treatment planning. TPSs adopting the Monte Carlo algorithm or the CCC algorithm both can precisely predict the real dose near the interface between air and human tissues when an extrapolated boundary is adopted, but the Monte Carlo algorithm is recommended for head and neck cancer radiotherapy.

## AUTHOR CONTRIBUTIONS

Hongbing Song and Jian Hu conceived and designed the study and Hongbing Song wrote the manuscript; Junxiang He and Guangdong Ma made the CT scan of the phantom in this study; Xiangpan Li and Lan Cheng assisted in data collection and analysis.

## CONFLICT OF INTEREST STATEMENT

The authors declare that there is no conflict of interest that could be perceived as prejudicing the impartially of the research reported.
